# Management of Very Early Small Cell Lung Cancer: A Canadian Survey Study

**DOI:** 10.3390/curroncol30070449

**Published:** 2023-06-23

**Authors:** Bayan Malakouti-Nejad, Sara Moore, Paul Wheatley-Price, David Tiberi

**Affiliations:** 1 Department of Radiology, Division of Radiation Oncology, University of Ottawa, 501 Smyth Road, Ottawa, ON K1H 8L6, Canada; dtiberi@toh.ca; 2 Department of Medicine, Division of Medical Oncology, University of Ottawa, 501 Smyth Road, Ottawa, ON K1H 8L6, Canada; smoore@toh.ca (S.M.); pwheatleyprice@toh.ca (P.W.-P.); 3 Ottawa Hospital Research Institute, 1053 Carling Avenue, Ottawa, ON K1Y 4E9, Canada

**Keywords:** small cell, practice patterns, local therapy, surgery, chemoradiotherapy

## Abstract

Concurrent chemoradiotherapy (CRT) is the standard of care for limited-stage small cell lung cancer (LS-SCLC). Local therapy—surgery or stereotactic body radiotherapy (SBRT)—with adjuvant chemotherapy may be appropriate for very early (T1-T2, N0) disease. There is variability in the management of these cases, which may lead to variability in patient outcomes. This study aimed to determine practice patterns for the management of very early LS-SCLC in Canada. A survey was developed and distributed to Canadian medical and radiation oncologists specialising in lung cancer. The survey consisted of three sections: (1) physician demographics, (2) general practice approach, and (3) preferred approach for three clinical scenarios (1: peripheral T1 lesion; 2: central T1 lesion; 3: peripheral T2 lesion). Responses were analysed to detect differences across cases and among physician groups. There were 77 respondents. In case 1, assuming medical operability, most respondents (73%) chose surgery and adjuvant chemotherapy, with 19% choosing CRT. CRT was selected by a higher proportion in case 2 (48%) and case 3 (61%) (*p* < 0.05). If medically inoperable, most chose CRT over local therapy in all cases, with more choosing CRT in case 2 (84%) and case 3 (86%) than in case 1 (55%) (*p* < 0.05). Subgroup analysis showed a predilection towards CRT in Western Canada and among more experienced physicians, and towards SBRT in Ontario. There is variability in the management of very early LS-SCLC in Canada. CRT remains the most popular strategy in most cases, with surgery preferred for small peripheral lesions. Larger and more central tumours are more likely to be managed with CRT. Variation in practice is correlated with region and physician experience. Our study illustrates the variability in the management of very early LS-SCLC in Canada and highlights the need for more robust investigations into the ideal approach for these patients.

## 1. Introduction

In Canada, lung cancer is the most prevalent cancer and the leading cause of cancer-related deaths. There were an estimated 30,000 new cases of lung cancer and 20,700 lung cancer deaths in Canada in 2022 [1]. Small cell lung cancer (SCLC), which accounts for approximately 15% of all lung cancers, is an aggressive neuroendocrine tumour with high metastatic potential [2]. The traditional staging method for SCLC is the Veterans Administration Lung Study Group (VALG) system, which categorises patients into either limited stage (LS-SCLC) or extensive stage (ES-SCLC) [3]. LS-SCLC is used to classify cases where all disease can be fully encompassed within a single radiotherapy field, while ES-SCLC refers to all other cases. Median survival for LS-SCLC is 12–16 months with treatment [4]. The standard of care in LS-SCLC is concurrent chemoradiation, specifically cisplatin and etoposide with thoracic radiotherapy starting with cycle 1 or 2 [5]. This is often followed by prophylactic cranial irradiation for the prevention of brain metastases [6].

Although the VALG staging system is still widely used in clinical practice, the American Joint Committee on Cancer (AJCC) TNM system has become more popular, partly due to its ability to further categorise the heterogeneous group of tumours that fall under the umbrella of LS-SCLC. Approximately 5% of LS-SCLC cases are T1 or T2, N0 by TNM staging at diagnosis [7], and may be referred to as very limited-stage small cell lung cancer (VLS-SCLC). Given their earlier stage, optimal management strategies for VLS-SCLC are uncertain. Retrospective data demonstrate favourable outcomes with local therapy, such as surgical resection or stereotactic body radiotherapy (SBRT), followed by adjuvant systemic therapy [8]. The National Comprehensive Cancer Network (NCCN) guidelines [9] recommend surgical management in the form of lobectomy and mediastinal lymph node sampling, followed by adjuvant systemic therapy, for surgical candidates. For those who are not medically operable, the guidelines suggest concurrent chemoradiation or SBRT followed by adjuvant systemic therapy.

The use of local therapy for LS-SCLC has increased in clinical practice. A National Cancer Database (NCDB) study [10] conducted in the United States in 2017 showed that the use of definitive surgery for patients with stage I SCLC increased from 14.9% of cases in 2004 to 28.5% in 2013. Similarly, the use of SBRT increased from 0.4% to 6.4% over that time period. Of the patients who received SBRT in this analysis, less than half (45.6%) received systemic therapy either before or after their radiotherapy. Given these trends, there is likely a great deal of variability in the way VLS-SCLC is managed in Canada. A survey of Canadian medical and radiation oncologists specialising in the treatment of lung cancer was conducted with the purpose of establishing current practice trends across the country in relation to VLS-SCLC.

## 2. Materials and Methods

A survey (Appendix A) was developed by the research team, which included a radiation oncologist and a medical oncologist, to explore physician approaches and experiences with very-early-stage (T1-T2, N0) LS-SCLC. The survey was divided into three sections: (i) physician demographics, (ii) experience with and approach to very-early-stage (T1-T2, N0) SCLC, and (iii) clinical scenarios. The clinical scenarios comprised three cases, each with different tumour characteristics: case 1 described a peripheral T1 lesion, case 2 illustrated a central T1 lesion, and case 3 was a peripheral T2 lesion. Respondents were asked to answer a set of questions for each case, including their ideal management strategy assuming medical operability or inoperability of the patient. The survey design was intended to identify the tumour and patient factors considered by physicians when deciding on treatment in these cases. Differences in therapy choices between cases 1 and 2 would indicate that tumour location is an important factor taken into consideration, and differences between cases 2 and 3 would suggest that tumour size is a variable considered.

Canadian medical and radiation oncologists specialising in the management of lung cancer were identified and contacted through their association with the Canadian Association of Radiation Oncology (CARO) or the Canadian Association of Medical Oncologists (CAMO), or individually by the study investigators.

The survey was conducted online using SurveyMonkey^®^ (www.surveymonkey.com, accessed on 19 December 2022, an online survey distribution and response collection tool, and took place between May and July 2022. Potential respondents were invited to participate through email, and the data were collected in an anonymous fashion. Local research ethics board approval was obtained prior to study commencement.

All survey responses were recorded in a spreadsheet (Microsoft Excel^®^) for analysis. Categorical variables were summarised using numbers and proportions. Chi-square test or Fisher’s exact test was used to compare proportions between independent groups when appropriate. Generalised linear mixed models were fit to compare the choices made in different cases because treatments selected by a single physician across all cases were considered to be correlated data. A *p*-value < 0.05 was considered statistically significant. All analyses were performed using SAS version 9.4 for Windows by SAS Institute Inc., Cary, NC, USA.

The data from the clinical scenarios were analysed to determine if tumour size or tumour location influenced the choice of therapy, and if there were any significant differences in practice between various physician groups—medical vs. radiation oncologists, academic vs. community physicians, physicians with low (<25%) vs. moderate (25–50%) vs. high (>50%) proportion of lung cancer patients, by region of the country (Eastern Canada, Quebec, Ontario, Western Canada), and by length of time in practice (<5 years, 5–10 years, 11–20 years, >20 years).

## 3. Results

There were 77 respondents to the survey. Of these, 70 (91%) completed the survey in its entirety. The data presented henceforth include all responses obtained for each individual question.

Of the 77 respondents, 30 were medical oncologists (39%), 46 were radiation oncologists (60%), and 1 response was received from a pulmonologist. Moreover, 16 respondents (21%) had practices with less than 25% lung cancer patients, 28 (36%) had practices with 25–50% lung cancer, and 33 (43%) had practices consisting of more than 50% lung cancer. Of physicians who reported their region of practice, 7 (9%) worked in Eastern Canada, 22 (29%) worked in Quebec, 23 (30%) worked in Ontario, and 24 (32%) worked in Western Canada. Most physicians (53/77, 69%) practiced in an academic setting rather than a community setting. Respondents also reported the length of time they had spent in practice. Physician characteristics are shown in Table 1.

Most respondents (68/76, 89%) did not have an institutional policy regarding the optimal management of this subset of patients, and most (68%, 51/75) were not aware of published guidelines regarding the optimal management of this group. Of those who reported being aware of guidelines, the National Comprehensive Cancer Network (NCCN) Clinical Practice Guidelines [9] were the most commonly referenced (16/24, 67%). Other major organisations whose guidelines were cited included the European Society for Medical Oncology (ESMO) [11], Cancer Care Ontario (CCO) [12], and Alberta Health Services (AHS) [13]. Respondents reported that these cases are discussed at tumour boards most of the time, with 54% (41/76) indicating they ‘always or almost always’ do so, while only 8% (6/76) indicated they ‘rarely or never’ do. These data are summarised in Table 2.

The survey asked physicians about their usual recommendations for the management of VLS-SCLC. Thirty percent (23/76) of respondents said they typically recommend concurrent chemoradiation, while thirty-eight percent (29/76) answered with surgery followed by adjuvant chemotherapy. Twenty-five percent (19/76) of respondents did not have a usual recommended therapy for these cases. Over half of the respondents (38/75, 51%) reported that the size of the lesion (T1 vs. T2) affects their choice of therapy. When asked if they pursue invasive mediastinal staging before deciding on management, 45% (33/74) said ‘always or almost always’, 43% (32/74) said ‘sometimes’, and 12% (9/74) said ‘rarely or never’. In terms of offering prophylactic cranial irradiation (PCI), 33% (16/48) of respondents said ‘always or almost always’, 40% (19/48) said ‘sometimes’, and 27% (13/48) said ‘rarely or never’. Data regarding physicians’ general approach to these cases are shown in Table 2.

Table 3 summarises the responses to three clinical scenarios. In general, physicians were more likely than not to recommend invasive mediastinal staging in all three cases, with the proportion ranging from 58% to 70% from case 1 to case 3. For medically operable patients, the most popular management options by far were concurrent chemoradiation and surgery followed by adjuvant chemotherapy. The proportion of respondents choosing chemoradiation increased over the three cases (19% in case 1, 48% in case 2, 61% in case 3), whereas the proportion choosing surgery and adjuvant chemotherapy decreased (73% in case 1, 47% in case 2, 36% in case 3). For the same scenarios, this time with medically inoperable patients, the most common management strategy in all three cases was chemoradiation (55%, 84%, and 86%). In case 1, 43% recommended SBRT followed by adjuvant chemotherapy, whereas only 15% and 14% recommended it in cases 2 and 3, respectively. Lastly, physicians were more likely to offer prophylactic cranial irradiation in all three cases, with the proportion ranging from 62% to 68%.

**Figure 1:**
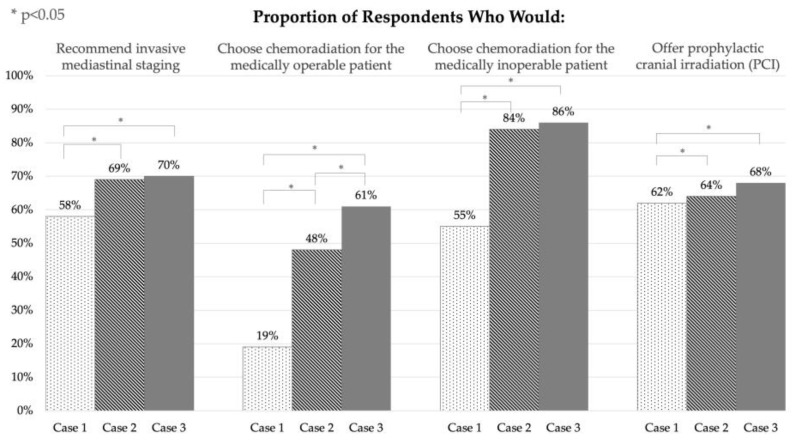
Comparison of physicians’ responses by case.

Generalised linear mixed models were performed to compare the decision-making patterns of physicians across the three presented cases. For the purpose of this analysis, the choice of concurrent chemoradiation was compared to the choice of any other option, i.e., local therapy (surgery or SBRT) with or without adjuvant chemotherapy. These analyses are illustrated in Figure 1.

Physicians were found to be significantly more likely to recommend invasive mediastinal staging in case 2 (central T1 lesion) and case 3 (peripheral T2 lesion) compared to case 1 (peripheral T1 lesion) (*p* < 0.05). Concurrent chemoradiation was significantly more likely to be recommended for medically operable patients both with a central location (case 2 vs. case 1, *p* < 0.05) and with a larger tumour (case 3 vs. case 2, *p* < 0.05). Comparing case 3 to case 2, physicians were more likely to choose chemoradiation in the case of a peripheral T2 lesion (case 3) than for a central T1 lesion (case 2) (*p* < 0.05). Similarly, for the medically inoperable patient, physicians were significantly more likely to recommend concurrent chemoradiation for both the more centrally located tumour (case 2 vs. case 1, *p* < 0.05) and the larger tumour (case 3 vs. case 1, *p* < 0.05). However, there was no difference between case 2 and case 3. Regarding prophylactic cranial irradiation, there was a nonsignificant trend towards offering PCI in case 2 vs. case 1 (*p* = 0.23), but significantly more physicians offered it in case 3 (peripheral T2 lesion) than in case 1 or 2 (*p* < 0.05).

Statistical analyses were also performed using generalised linear mixed models to detect disparities in practice between different physician cohorts. These analyses are summarised in Table 4.

There were no statistically significant differences in recommendations between medical oncologists and radiation oncologists, physicians with different percentages of their practice consisting of lung cancer patients, or physicians working in academic versus community settings. However, significant differences were observed in treatment choices based on geographic location. Physicians in Western Canada were more likely to recommend concurrent chemoradiation, while those in Ontario were more likely to opt for local therapy (surgery or SBRT followed by adjuvant chemotherapy). There was also a difference in management strategy based on length of time in practice. Physicians with 11 or more years of practice were more likely to choose concurrent chemoradiation compared to their counterparts with 10 or fewer years of experience.

## 4. Discussion

The results of our survey show that there is significant variation in the management of very early (T1-T2, N0) SCLC by lung cancer specialists in Canada. If the patient is deemed medically operable, most physicians would opt for either concurrent chemoradiation or surgery followed by adjuvant chemotherapy (Table 3). In terms of factors that influence decision making, the responses to the clinical scenarios suggest that larger tumours—and, to a lesser extent, more central tumours—are more likely to be treated with concurrent chemoradiation (Figure 1). When a patient is not suitable for surgical resection, most respondents recommend either concurrent chemoradiation or SBRT followed by adjuvant chemotherapy. However, across all three clinical scenarios, SBRT was chosen less frequently than surgery for the medically operable patient (Table 3). This preference may be because surgery allows for the sampling of mediastinal lymph nodes. While our survey did not specify the surgical technique, lobectomy and mediastinal lymph node sampling are recommended for early SCLC [9]. Respondents were more inclined to recommend concurrent chemoradiation—and thus less likely to recommend SBRT and adjuvant chemotherapy—for more central and larger tumours (Figure 1).

Most of the participants suggested performing invasive mediastinal staging before definitive treatment, and this proportion increased both for more centrally located and for larger tumours. The majority of respondents also suggested offering prophylactic cranial irradiation (PCI) in all three clinical scenarios. The likelihood of offering PCI increased with increasing tumour size (Figure 1).

We noted a few identifiable physician characteristics that were associated with a predilection towards a particular treatment strategy. Physicians practicing in Western Canada (BC, AB, SK, MB) were more inclined than others to recommend concurrent chemoradiation for medically operable patients, while physicians in Ontario were more likely than others to recommend SBRT and adjuvant chemotherapy for medically inoperable patients. The reason for this regional variation is uncertain, but it may be due to differences in institutional culture. Further inquiry may be warranted. Furthermore, physicians who had been in practice longer (more than 10 years) were more likely than newer physicians to recommend concurrent chemoradiation in both operable and inoperable patients. This may be because the evidence supporting local therapy for these patients is recent, and uptake has been slower among physicians who have spent longer in practice and have established management patterns.

Based on a review of the relevant literature, several key recommendations can be made regarding the management of very early LS-SCLC:

1. Multidisciplinary discussion is important in cases where prospective evidence is limited. These cases should be discussed at multidisciplinary tumour boards to determine the most appropriate management strategy for each individual patient, based on their unique characteristics and care goals of care.
2. Invasive mediastinal staging should be performed prior to deciding on a treatment strategy [9], as occult nodal disease is not uncommon in SCLC. Studies have shown that among patients who underwent surgery, those who had four or more lymph nodes removed had better outcomes than those with fewer than four nodes removed, likely due to more accurate staging prior to treatment [14].
3. Surgery followed by adjuvant chemotherapy is a viable approach for medically operable patients, particularly for T1 lesions, as recommended by guidelines [9,11]. However, caution should be exercised for larger lesions. On subgroup analysis in one study evaluating the benefit of surgery for SCLC, T2 lesions had poorer survival and higher unresectability rates than T1 lesions [15].
4. For patients who are not medically operable, SBRT followed by adjuvant chemotherapy may be considered [16]. The evidence for this recommendation is not strong, based on a few small retrospective studies. The largest of these, published by Verma et al., included 74 patients and showed overall survival rates comparable to those in previously published surgical series [17]. However, outcomes were worse with tumours larger than 2 cm, indicating that this approach is best suited for smaller (T1) lesions.
5. MRI surveillance is a reasonable alternative to prophylactic cranial irradiation (PCI) in this population. The original meta-analysis regarding PCI in patients with SCLC [18] included older studies with less accurate staging. As a result, patients included were at higher risk, with over 10% having extensive-stage disease. In a retrospective review of patients with SCLC who underwent resection, PCI had survival benefits for stage II and stage III disease but not for stage I disease [19]. Given the relatively lower risk of brain metastasis in this population and the neurotoxic effects of cranial irradiation, the risk likely outweighs the benefit.

This study has several strengths. First, the survey was widely distributed among physicians across the country, ensuring representation from all regions. Second, the clinical scenarios presented in the survey were well designed to highlight differences in practice patterns based on tumour characteristics. Third, the survey explicitly differentiated between medically operable and inoperable patients, which is important given that a significant proportion of patients with SCLC are not candidates for surgery.

There are a few limitations to this study that should be considered. Firstly, the sample size was relatively small (n = 77) in comparison to the number of medical and radiation oncologists who treat lung cancer in Canada. Therefore, making direct comparisons between different groups may be challenging. Additionally, our survey included three clinical scenarios: a peripheral T1 lesion, a central T1 lesion, and a peripheral T2 lesion. To better determine the impact of tumour characteristics on management decisions, it may have been helpful to include a fourth case involving a central T2 lesion. Lastly, although our survey referred to surgery, it did not specify the surgical technique. It may have been beneficial to mention that surgery would consist of lobectomy with mediastinal lymph node sampling. However, it is uncertain whether this would have influenced the results.

The present study provides insight into the management of very early LS-SCLC in Canada. Subsequent research could focus on assessing outcomes associated with the different management strategies in use across the country, using data from provincial databases or single-institution chart reviews.

## 5. Conclusions

There is currently a lack of consensus among Canadian lung cancer specialists regarding the optimal management of very early (T1-T2, N0) SCLC patients, and this may lead to variation in patient outcomes based on the choice of therapy. Medically operable patients may receive concurrent chemoradiation or surgery with adjuvant chemotherapy, while medically inoperable patients may undergo chemoradiation or SBRT with adjuvant chemotherapy. The lack of level I evidence to support one approach over the other contributes to this lack of consensus. Generally, physicians tend to recommend local therapy for smaller peripheral lesions. There are differences in practice patterns depending on the region of the country and time in practice. This study helps establish the current management of very early LS-SCLC in Canada and how it varies based on patient and physician factors. This will hopefully help encourage discussion regarding this relatively small patient population and the fact that they may benefit from an alternative treatment approach to other cases of LS-SCLC. A multidisciplinary decision-making process is warranted. As more information on outcomes with different approaches becomes available, this can help refine the optimal treatment strategy for these patients and reduce variability in their care.

## Figures and Tables

**Table 1:** Respondent demographics.

**Specialty (n = 77)**	
Medical oncologist	30 (39%)
Radiation oncologist	46 (60%)
Other	1 (1%)
**Percentage of practice involving lung cancer patients (n = 77)**	
<25%	16 (21%)
25–50%	28 (36%)
>50%	33 (43%)
**Region of practice (n = 76)**	
Eastern Canada (NB, NS, NL, PE)	7 (9%)
Quebec (QC)	22 (29%)
Ontario (ON)	23 (30%)
Western Canada (BC, AB, SK, MB)	24 (32%)
**Nature of practice (n = 77)**	
Majority (>50%) academic	53 (69%)
Majority (>50%) community	24 (31%)
**Length of time in practice (n = 77)**	
<5 years	12 (16%)
5–10 years	19 (25%)
11–20 years	24 (31%)
>20 years	22 (29%)

**Table 2:** Physicians’ experience with and general approach to very early (T1-T2, N0) SCLC.

**Presence of institutional policy regarding optimal management of this group (n = 76)**	
Yes	8 (11%)
No	68 (89%)
**Awareness of guidelines regarding optimal management of this group (n = 75)**	
Yes	24 (32%)
No	51 (68%)
**Are these patients discussed at tumour boards? (n = 76)**	
Always or almost always	41 (54%)
Sometimes	29 (38%)
Rarely or never	6 (8%)
**Usual recommendation for management (n = 76)**	
Concurrent chemoradiation	23 (30%)
Surgery alone	2 (3%)
Surgery with adjuvant chemotherapy	29 (38%)
SBRT alone	0 (0%)
SBRT with adjuvant chemotherapy	3 (4%)
No standard, depends on the case	19 (25%)
**Does the size of the lesion (T1 vs. T2) affect your recommendation? (n = 75)**	
Yes	38 (51%)
No	37 (49%)
**Do you pursue invasive mediastinal staging? (n = 74)**	
Always or almost always	33 (45%)
Sometimes	32 (43%)
Rarely or never	9 (12%)
**Do you offer prophylactic cranial irradiation? (n = 48)**	
Always or almost always	16 (33%)
Sometimes	19 (40%)
Rarely or never	13 (27%)

**Table 3:** Physicians’ responses to clinical scenarios.

**Would you recommend/pursue invasive mediastinal staging?**			
	Case 1—peripheral T1 (2 cm) lesion (n = 74)	Case 2—central T1 (2 cm) lesion (n = 74)	Case 3—peripheral T2 (4.5 cm) lesion (n = 74)
Yes	43 (58%)	51 (69%)	52 (70%)
No	31 (42%)	23 (31%)	22 (30%)
**The patient was deemed to be medically operable. What is your recommended management?**			
	Case 1—peripheral T1 (2 cm) lesion (n = 73)	Case 2—central T1 (2 cm) lesion (n = 73)	Case 3—peripheral T2 (4.5 cm) lesion (n = 74)
Concurrent chemoradiation	14 (19%)	35 (48%)	45 (61%)
Surgery alone	3 (4%)	2 (3%)	1 (1%)
Surgery with adjuvant chemotherapy	52 (73%)	34 (47%)	27 (36%)
SBRT alone	0 (0%)	0 (0%)	0 (0%)
SBRT with adjuvant chemotherapy	3 (4%)	2 (3%)	1 (1%)
**The patient was deemed to be medically inoperable. What is your recommended management?**			
	Case 1—peripheral T1 (2 cm) lesion (n = 74)	Case 2—central T1 (2 cm) lesion (n = 74)	Case 3—peripheral T2 (4.5 cm) lesion (n = 73)
Concurrent chemoradiation	41 (55%)	62 (84%)	63 (86%)
SBRT alone	1 (1%)	1 (1%)	0 (0%)
SBRT and adjuvant chemotherapy	32 (43%)	11 (15%)	10 (14%)
**Would you offer prophylactic cranial irradiation (PCI)?**			
	Case 1—peripheral T1 (2 cm) lesion (n = 73)	Case 2—central T1 (2 cm) lesion (n = 74)	Case 3—peripheral T2 (4.5 cm) lesion (n = 72)
Yes	45 (62%)	47 (64%)	49 (68%)
No	28 (38%)	27 (36%)	23 (32%)

**Table 4:** Comparison of physicians’ management plan by subgroup.

Physician Subgroup	Proportion Who Chose Concurrent Chemoradiation for the Medically Operable Patient			Proportion Who Chose Concurrent Chemoradiation for the Medically Inoperable Patient		
	Case 1	Case 2	Case 3	Case 1	Case 2	Case 3
**Specialty**						
Medical oncologists	27%	50%	67%	60%	83%	93%
Radiation oncologists	14%	48%	56%	51%	84%	81%
*p*-value	*0.1906*	*0.8420*	*0.3512*	*0.4554*	*1.0000*	*0.1776*
**Percentage of practice lung cancer**						
<25%	27%	53%	80%	60%	80%	87%
25–50%	14%	44%	61%	54%	93%	86%
>50%	20%	48%	52%	55%	77%	87%
*p*-value	*0.6101*	*0.8566*	*0.1810*	*0.9184*	*0.2455*	*1.0000*
**Region of practice**						
Eastern (NB, NS, NL, PE)	14%	33%	71%	71%	71%	86%
Quebec (QC)	5%	45%	60%	60%	90%	85%
Ontario (ON)	9%	35%	44%	30%	70%	77%
Western (BC, AB, SK, MB)	39%	65%	74%	70%	96%	96%
*p*-value	0.0206 *	*0.1731*	*0.2041*	0.0360 *	*0.0598*	*0.3067*
**Nature of practice**						
Majority (>50%) academic	20%	45%	59%	51%	84%	88%
Majority (>50%) community	17%	55%	65%	65%	83%	83%
*p*-value	*1.0000*	*0.4585*	*0.6020*	*0.2541*	*1.0000*	*0.7153*
**Length of time in practice**						
<5 years	0%	30%	40%	20%	70%	60%
5–10 years	5%	37%	63%	47%	74%	79%
11–20 years	30%	58%	67%	63%	88%	92%
>20 years	29%	55%	62%	71%	95%	100%
*p*-value	0.0456 *	*0.2999*	*0.5286*	0.0413 *	*0.1413*	0.0091 *

* *p*-value < 0.05.

## Data Availability

No new data were created.

## Appendix A

SURVEY—MANAGEMENT OF VERY EARLY (T1-T2, N0) SMALL CELL LUNG CANCER

SECTION I: RESPONDENT DEMOGRAPHICS

1 What is your specialty?
  a Medical Oncologist
  b Radiation Oncologist
  c Other

2 What percentage of your practice involves lung cancer patients?
  a Less than 25%
  b 25–50%
  c More than 50%

3 Which region of the country do you practice in?
  a Eastern Canada (NB, PE, NS, NL)
  b Quebec
  c Ontario
  d Western Canada (BC, AB, SK, MB)

4 What is the nature of your practice?
  a Majority (>50%) academic
  b Majority (>50%) community

5 How long have you been in practice?
  a Less than 5 years
  b 5–10 years
  c 11–20 years
  d More than 20 years

6 If you are a Medical Oncologist, does your centre have Radiation Oncology services?
  a Yes
  b No

SECTION II: APPROACH TO EARLY LIMITED-STAGE SCLC (T1-T2, N0)

1 What is your usual recommendation for treating early limited-stage SCLC?
  a Chemoradiation
  b Surgery alone
  c Surgery followed by adjuvant chemotherapy
  d SBRT alone
  e SBRT followed by adjuvant chemotherapy
  f No standard, depends on the case

2 Does your centre have an institutional policy regarding management of these cases?
  a Yes
  b No

3 Are you aware of any guidelines regarding management of these cases?
  a Yes
  b No

4 If yes, please indicate the guideline: ______________

5 Approximately what proportion of your SCLC cases are these early (T1 or T2, N0) cases?
  a Less than 10%
  b 10–30%
  c 30–50%
  d Greater than 50%

6 Do these patients get discussed at tumour boards?
  a Always or almost always
  b Sometimes
  c Rarely or never

7 Do you pursue invasive mediastinal staging in these cases, if not already done?
  a Always or almost always
  b Sometimes
  c Rarely or never

8 Does the size of the tumour (T1 vs. T2) influence how you manage these cases?
  a Yes
  b No

9 If you offer SBRT, what fractionation do you use?
  a Same fractionation as NSCLC cases
  b Different fractionation than NSCLC cases

10 Do you routinely offer these patients prophylactic cranial irradiation (PCI)?
  a Always or almost always
  b Sometimes
  c Rarely or never

11 Do you routinely offer these patients chemotherapy in addition to local therapy, assuming no contraindications to systemic therapy?
  a Always or almost always
  b Sometimes
  c Rarely or never

SECTION III: CLINICAL SCENARIOS

CASE 1: You meet a 65-year-old male in consultation with a 2.0 cm (T1) small cell lung cancer. The tumour is located in the periphery of the right lower lobe. PET scan is negative for any nodal involvement. The patient is deemed fit enough to receive chemotherapy and radiotherapy.

1 Would you recommend/pursue invasive mediastinal staging?
  a Yes
  b No

2 The patient was seen by a surgeon and deemed to be medically operable. What is your recommended management?
  a Concurrent chemoradiation
  b Surgery alone
  c Surgery followed by adjuvant chemotherapy
  d SBRT alone
  e SBRT followed by adjuvant chemotherapy

3 The patient was seen by a surgeon and was deemed medically inoperable. What is your recommended management?
  a Concurrent chemoradiation
  b SBRT alone
  c SBRT followed by adjuvant chemotherapy

4 Would you offer the patient prophylactic cranial irradiation (PCI)?
  a Yes
  b No

CASE 2: You meet a 65-year-old male in consultation with a 2.0 cm (T1) small cell lung cancer. The tumour is located centrally in the right lower lobe, adjacent to the hilum. PET scan is negative for any nodal involvement. The patient is deemed fit enough to receive chemotherapy and radiotherapy.

1 Would you recommend/pursue invasive mediastinal staging?
  a Yes
  b No

2 The patient was seen by a surgeon and deemed to be medically operable. What is your recommended management?
  a Concurrent chemoradiation
  b Surgery alone
  c Surgery followed by adjuvant chemotherapy
  d SBRT alone
  e SBRT followed by adjuvant chemotherapy

3 The patient was seen by a surgeon and was deemed medically inoperable. What is your recommended management?
  a Concurrent chemoradiation
  b SBRT alone
  c SBRT followed by adjuvant chemotherapy

4 Would you offer the patient prophylactic cranial irradiation (PCI)?
  a Yes
  b No

CASE 3: You meet a 65-year-old male in consultation with a 4.5 cm (T2) small cell lung cancer. The tumour is located in the periphery of the right lower lobe. PET scan is negative for any nodal involvement. The patient is deemed fit enough to receive chemotherapy and radiotherapy.

1 Would you recommend/pursue invasive mediastinal staging?
  a Yes
  b No

2 The patient was seen by a surgeon and deemed to be medically operable. What is your recommended management?
  a Concurrent chemoradiation
  b Surgery alone
  c Surgery followed by adjuvant chemotherapy
  d SBRT alone
  e SBRT followed by adjuvant chemotherapy

3 The patient was seen by a surgeon and was deemed medically inoperable. What is your recommended management?
  a Concurrent chemoradiation
  b SBRT alone
  c SBRT followed by adjuvant chemotherapy

4 Would you offer the patient prophylactic cranial irradiation (PCI)?
  a Yes
  b No

END OF SURVEY
